# The influence of successor’s implicit power and explicit power on dual innovation———The mediating effect of board dissent

**DOI:** 10.1371/journal.pone.0275603

**Published:** 2022-11-14

**Authors:** Wang Feifei, Wu Jiong, Marcello Russo, Luyang Gao

**Affiliations:** 1 School of Management, Hangzhou Dianzi University, Hangzhou, China; 2 The Glorious Sun School of Business Management, Donghua University, Shanghai, China; 3 Business School, University of Bologna, Bologna, Emilia, Romagna, Italy; 4 Cardiff Business School,Cardiff University, Cardiff, United Kingdom; Sri Eshwar College of Engineering, INDIA

## Abstract

This study explores the influence of the power of family business successors on firm innovation under the theory of social embeddedness. Based on the 2000–2019 unbalanced panel data of listed Chinese family enterprises, this study empirically examines the differences in the influence of the implicit and explicit power of successors on incremental and radical innovation respectively. Our findings show that explicit power has a more positive impact on incremental innovation, while implicit power is more conducive to promoting radical innovation. In addition, the study finds that the reason why the explicit power of succession does not have a significant impact on radical innovation, that is, the reason why board dissent is not related to radical innovation, is that some of the major innovation decisions in the enterprise are not all made at formal meetings. The research conclusions not only extend the theoretical application of social embeddedness in family enterprises, but also provide certain practical guidance for promoting enterprise innovation.

## Introduction

In the next five to ten years, about 3 million Chinese family business companies is projected to face successionchange of leadership which means that China will enter an intensive period of family business inheritance [1]. Under the current economic and social situation, innovation is a key to promote a family enterprise to become sustainable in order to sustain company’s performance [2,3]. Empirical evidence has supported that managerial power is an important factor in the process of strategic decision-making to propel innovation [4]. Managers with high power may have greater freedom in decision-making, meaning that the constraints in the decision-making process need to be reduced to prevent innovation limitation [5]. Marginson & Mcculay (2008) [6] believe that the higher the centralization of managers’ power is, the more beneficial it is to develop R&D strategies that enable sustainable competitive advantages, so as to improve enterprises‘ innovation capability. Eggers & Kaplan (2009) [7] found that CEOs with greater power tend to have more resources and greater influence, and tend to play a more powerful role in the development of new products. Obviously, the relationship between the power of enterprise managers and organizational innovation behaviors has been explored by many scholars [4–6]. In an increasingly competitive market, the power of the successors of the family entrepreneur becomes the key to improve the innovation decision of the enterprise, as they are the top decision maker of the enterprise. However, there are still two limitations in current research.

First is the lack of dual causal exploration. Individual power can come from the legal granting of formal authority from higher authorities, i.e., explicit power, or from some non-institutional arrangement, i.e. implicit power [8]. Since the influence of different power on technological innovation of enterprises is different [9], it needs to be distinguished according to different situations. According to the dual innovation theory, incremental innovation and radical innovation have different impact in terms of risks [10]. Studies on antecedent variables of ambidextrous innovation show that the influence of CEO power on incremental innovation and radical innovation is also different [11]. However, most of existing research has focused on only one face of the coin, either focusing on the influence of a single source of power on dual innovation [12], or examining the effects of different powers on a single type innovation, either incremental or radical innovation [11], neglecting the difference in the influence of different sources of power on different types of innovation.

Secondly, there is a lack of exploration of the mechanism of this effect. Social embeddedness theory has shown that human beings do not act in isolation outside their social context, but instead, human actions are closely embedded in social relations and within a social structure [1]. In this stream of research, power has been considered as a core concept in the construction of interpersonal relationships [13]. It is embedded in a certain historical and social structure including formal and informal system that reflects the beliefs of social members [14]. Specifically, interpersonal interaction is important because people consider their interpersonal relationships with others as a significant factor facilitating or obstructing the pursuit of economic and social goals. In the same vein, the actions of successor will be affected by the interpersonal relationship in the context of family business, through which they can gain the support and power from existing managers [15]. A large number of studies have shown that high-quality interpersonal relationships produce a stronger tolerance for failure, which provides an appropriate environment for organizational innovation and thus improves the innovation level of listed companies [16]. However, there is less literature exploring the impact of the power held by a successor on innovation from the perspective of interpersonal interaction, which needs to be further explored.

Based on the Corporate Governance Theory, it is necessary to pay attention to the interaction between actors in order to deeply understand the decision-making behavior of enterprises. The introduction of existing managers’ constraint effect on successors can make a more comprehensive exploration of the influence of successors’ power perspective on family business innovation. In addition, board membership is an important consideration,, as board members have the right to challenge the innovation decisions of their successors [17]. Some scholars pointed out that the board of directors is like a "rubber-stamp" and a "puppet" of the management, meaning that they are in a passive position to raise objections [18], so formal board meetings may become the role of "vase". However, there is still a lack of in-depth discussion on the role of board dissent in the decision-making process of enterprises. Therefore, according to the Corporate Governance Theory [19] and decision-making process of the board of directors, this study examines the interpersonal interaction between the successors and board members to explore the impact of decision-making process on innovation.

Based on the above questions, this study explores the impact of the authority of the successor of a family business on corporate innovation. Compared with previous studies, this study contributes to empirically examine the influence of implicit power and explicit power on dual innovation respectively. Secondly, it takes into account process factors and explores the mediating effect of board dissent between managerial power and corporate innovation. To this end, this study determines the kinship relationship between the parent entrepreneur and the successor of the family enterprise according to the public information such as the annual report of listed companies, the Baidu search engine and the news report, and selects the A-share listed family enterprise from 2001 to 2019 for empirical testing.

## Theoretical analysis and research hypothesis

### Manager power and enterprise innovation

Managers have a crucial role in supporting innovative behaviors and their impetus to innovation is often the driving force to organizational evolution. Research has found that managers with greater power tend to have more resources and influence and, therefore, they are more capable of playing an influential role in the development of new products or services [7]. The current research on managerial power and enterprise innovation mainly focuses on two categories, as shown in Table 1. The first focuses on the relationship between single power and dual innovation of the enterprise, focusing on whether and how manager power affects the different innovation activities of the organization. The second type focuses on the relationship between multidimensional power and enterprise innovation, focusing on what the power of managers is based on to have an impact on enterprise innovation. Therefore, there is still limited research on the mechanism between the power of managers and the innovation of enterprises.

**Table 1:** Summarizes the main literature on manager power and enterprise innovation.

Category	Representative Authors	Contents
I	Sariol & Abebe [11]	The impact of CEO power on radical and incremental innovation is examined.
	Xu, Xiao, & Zhou [20]	The regulatory effect of family authority in second-generation succession and dual innovation.
II	Tan, &Chen [12]	From the perspective of the three subdivision powers of financial power, administrative power and personnel power, the mechanism of power allocation on innovation is discussed.
	Hu [9]	The moderating effect of structural power and reputational power on technological innovation investment and corporate performance.

### The difference between the influence of implicit power and explicit power on dual innovation

Explicit power mainly comes from the organizational structure and assignment of the power based on the role in the hierarchical structure. It is the formal, legal and institutional basis of power in an organization. It emphasizes that subordinates should obey their superiors based on their different role in the hierarchy. The implicit power on the other hand comes from informal factors, such as the influence formed by the individual’s ability, knowledge and morality in the group, and it can influence decision-making and implementation.

Incremental innovation mainly emphasizes the expansion of the current market share and product market portfolio through simple adjustments and minor changes, as well as the introduction of new products and services, and the expansion of company scale to meet new market target needs [21]. Incremental innovation has a lower investment risk than radical innovation and it may bring benefits to both successors and executive managers of a family business in the short term. Therefore, we contend that the interpersonal relationships between the different parties involved in the decision-making process have different degrees of power, will not cause conflicts between the successor and board members in decision-making in incremental innovation activities. The reason is that people will more likely to support each other when they have same interests, as it could be in the case of incremental innovation that can bring short-term benefits for all [13].

According to the social embeddedness theory, power implies the control of scarce resources [22]. From this sociological perspective, the interpersonal relationship maintained by the subordinate and the superior is related to the resource management ability of the superior [13]. Therefore, when superiors and subordinates reach an agreement on the implementation of an innovation project, explicit power could be more conducive to mobilizing the human, material and financial resources needed for incremental innovation, showing stronger influence on incremental innovation than implicit power. For example, the final decision is made in the interaction between the successor and the senior members as the company increases its R&D investment. The explicit power possessed by the successor can directly make decisions to meet such needs quicker, while relying on implicit power would require a series of tedious tactics and evaluations to achieve the purpose of increasing research expenses instrumental for incremental innovation. Based on this, this study puts forward the following hypotheses:

H1: Compared with implicit power, explicit power has a stronger positive influence on incremental innovation.

Radical innovation is an innovation that is more groundbreaking [11]. To meet the needs of new customers, companies need to acquire new knowledge to develop new products [10]. Such innovation bring novelty to the company’s market share by expanding its product line and exploring potential customers [23]. However, radical innovation has high risk for the company as it requires a long investment cycle, high exit costs, and it has a greater investment risk. Since the main goal of a family business is to maintain the inheritance of the family property and a long term development [24], it would like to take the high risk of long-return projects. However, the goal of the family business might collide with the ones of the board of directors for the reason that directors tend to concern more about personal income and job security leading them to prefer projects with quick returns and lower investment risks.

According to social embeddedness theory, if power is not linked to the possession of scarce resources, it means that whoever has the right to speak determines the direction of public opinion [22]. From this perspective, the interpersonal relationship maintained by the subordinate and the superior is related to the value rationality behavior motivation [13]. Implicit power can act on subordinates to make them follow voluntarily for the reason that such behavior is generally not the control of resources, but the spiritual belief, worship, identification and appreciation with the leader [13]. Therefore, when successors make radical innovation decisions based on explicit power, the ability of directors to perceive uncertainty will be increased, which will affect the investment of radical innovation. On the contrary, when a radical innovation decision is made based on implicit power, the directors assume that the behavior of the successor is reasonable, so that they will actively support the radical innovation decision of the successor. Based on this, this study puts forward the following hypotheses:

H2: Compared with explicit power, implicit power has a stronger positive impact on radical innovation.

### The mediating role of board’s dissent

Board meetings are the most important place for directors to collect information, supervise management, and implement decisions [25] where the formulation of strategies, changes in executives, and other resolutions are the results of collective decisions [19]. When the board of directors makes collective decisions, the board members mainly adopt the voting system for the company’s proposals which mainly includes three kinds of opinions: "agree", "against" and "abstention". According to China’s Company Law, whether a director’s decision is passed or not should follow the principle of "majority support". Therefore, "abstention" and "opposition" play the same role in China’s corporate governance practice and both express their disapproval to the decision [26]. According to the expectation states theory, the differences in the status of members in groups can affect internal dynamics [17]. Board informal hierarchy will affect the submissive behaviors among the members of the board, that is, the directors with low power tend to follow the directors with high power. In such a situation, directors are less likely to raise an objection when making decisions, and more likely to reach consensus. Therefore, we expect that the implicit power or explicit power of the successor is negatively correlated with the dissent of the board of directors.

In addition to the formal interaction such as the board meeting, the informal interaction formed by trustand rules among directors also plays an important role in the performance of functions of the board of directors [27]. Informal interactions between directors–such as phone calls, face-to-face meetings, e-mails, and banquets, etc.–are common and important to enterprise decisions [28]. This especially happens under the society of China where informal interaction based on kinship relationship plays a more influential role in social behavior due to the patriarchal clan culture system. Informal interactions therefore can make up for ineffective formal corporate governance systems [29].

Incremental innovation often occurs in the day-to-day decisions of formal meetings, and it can occur at every stage of the business’s operational process. Regardless of whether the successor has implicit or explicit power, board dissent has impact on incremental innovation. Compared with incremental innovation, radical innovation is characterized by both high returns and high risks, and this type of innovation usually happens with the major projects of enterprises [10]. The final decision of the non-routine innovation usually made in the discussion of the successor and the senior members (such as the chairman, general manager, financial director, etc.), and the opinions formed in the board meeting can only be used as a reference for the final decision. However, studies have shown that there are some rebels in social life such as colleagues, friends, and family members showing disagreement when everyone else is in agreement [30]. At present, most family businesses in China are in a critical period of succession. Due to the differences in different generation between senior executives and latter successors such as growth environment, knowledge structure and attitude towards life, they are likely to have cognitive differences, thus causing conflicts in business management and operation [15]. As a result, executive members often become rebels in the intergenerational succession of family businesses. Generally, the behavior motivations of successors and senior management members are not consistent. The successors hope to realize the long-term development of the family business by taking radical innovation [31], while the key directors may prefer to avoid radical innovation in order to obtain short-term benefits [32]. Only when the successor has implicit power and executive members have spiritual admiration for the successor, can the radical innovation proposals not be vetoed in private discussions. When the successor made decisions based on implicit power, the board of directors dissent has an impact on the incremental innovation. On the contrary, when the successor has explicit power, senior executive members may become rebels of radical innovation out of personal interests, thus leading to the objection of radical innovation. Therefore, there is no direct relationship between board dissent and radical innovation.

H3a: Board’s dissent plays an mediating role in the relationship between implicit power and incremental innovation.

H3b: Board’s dissent plays a mediating role in the relationship between implicit power and radical innovation.

H3c: Board’s dissent plays a mediating role in the relationship between explicit power and incremental innovation.

**Fig 1:**
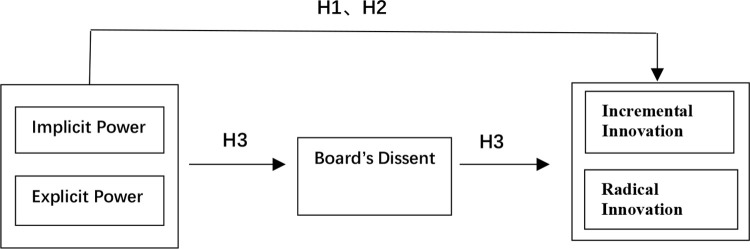
Research framework.

In summary, the research framework is shown in Fig 1.

## Research design

### Research sample

This study takes the Chinese family-listed companies from 2011 to 2019 as a research sample. In terms of the definition of family business, this paper refers to the research of Ellul et al. (2010) [33] and Wu et al. (2021) [1] A family enterprise is defined as: (1) the ultimate controlling shareholder of the enterprise can be traced back to a single natural person or family; (2) A natural person who is the ultimate controlling shareholder or the actual controller in the controlling family; (3) At least two family members who are related by kinship to the actual controller of the family are serving at the top level of the enterprise; (4) The family tries to get the next generation of members to continue the ownership of the business. On this basis, the CSMAR database "Database of Private Listed Companies" was used, and a sample of A-share listed family enterprises was selected based on the indicators of "name of actual controller" and "type of actual controller" because the indicators such as research expenditure and development capitalization expenditure by domestic listed family enterprises are not available. Therefore, this article checks the prospectus, the company’s annual report, Sina stock network and Juchao Information and other professional websites to check each other in multiple ways.

The research samples were excluded as follows: (1) St, *ST, S*ST and delisted samples were excluded; (2) Exclude the sample of incomplete succession, that is, the next generation of members of the parent entrepreneur (children, son-in-law, nephews, nieces) are not serving as the chairman or general manager of the listed company; (3) Eliminate missing data and abnormal samples; (4) Exclude samples listed after the succession of family business successors; To eliminate the effect of outliers, continuous variables are Winsorized at 1% above and below. Through the above process, we finally obtained 310 observed values of 85 A-share family enterprises of family enterprises with the period from 2011 to 2019.

### Dependent variable

Dual innovation includes incremental innovation and radical innovation. Drawing on the research of Bi et al. (2017) [34], the percentage of development capital expenditures to the total assets at the beginning of the year is used to measure incremental innovation, and the percentage of research expenditures to total assets at the beginning of the year is used to measure radical innovation.

### Independent variable

This study draws on the studies of La Porta et al. (1999) [35] calculating the proportion of the listed company’s control power (voting power) owned by the successor to represent the successor’s control right. The calculation is as follows:

$S_{0}=\sum_{i=1}^{n}\min_{i}(a_{i1},a_{i2},\ldots ,a_{ij})$ *a*_*ij*_∈(0.1), where “i” stands for control chain i and t stands for layer t on the control chain.

In existing studies, the main indicators for measuring explicit power are experience, education, and gender. Obviously, a single indicator cannot accurately measure the implicit power (Dom) of successors, and too many indicators are prone to multicollinearity. Based on the practice of Quan et al. (2010) [36], this study constructed a comprehensive index to reflect the implicit power of successors through principal component regression of five indicators including work experience, age, educational background, overseas study background and gender. Firstly, the data of working time (GTI), age (GAG), education background (GED), overseas study background (GEX) and gender (GSE) of the successors were standardized; Then, Bartlett test (P = 0.000) and KMO test (K = 0.719) were carried out for these indexes, and the test results showed that they were suitable for principal component analysis; Finally, a principal component (as shown in Table 2) is extracted based on the two criteria of feature root greater than 1 and cumulative contribution rate greater than 0.7. According to the factor scoring coefficient matrix, the first principal component can be expressed as a linear combination of variables: Dom = 0.671Gti+0.379Gag−0.084Ged−0.197Gex+0.640Gse.

**Table 2:** List of principal components.

Main ingredient	Characteristic root	Accumulation
1	1.436	71.168
2	0.864	81.287
3	0.723	88.914
4	0.657	90.347
5	0.551	100.000

### Intermediary variables

According to Shanghai and Shenzhen Stock Exchange regulations, board members with voting rights need to vote on relevant proposals which include approval, opposition, and abstention in the decision-making process of the board of directors of listed companies. According to Ma & Khanna (2016), voting opinions other than “agree” are regarded as dissenting opinions [37]. In this research, we contend that voting against board members is a way to express strong dissent, and abstention is also an expression of dissent. These two objections must reflect the interpersonal conflict between the successor and the directors in the corporate innovation decision-making, which will affect the approval of the board meeting. Therefore, the board members’ votes and abstentions are regarded as dissent from the board of directors. A dissent (Dis) from the board of directors during the year is marked as value 1, otherwise is 0.

### Control variables

With reference to existing research [34], this research uses company age, cash flow, growth, rate of return, institutional investment and shareholder equity as control variables. Among them, the age of the company is measured by the difference between the year of succession and the year when the company is listed. Cash flow is measured by net cash flow generated from operating activities, growth is measured by TobinQ, yield is measured by return on net assets, and institutional investment is measured by institutional investment. Shareholders’ equity is measured by year-end shareholders’ equity. In addition, in order to eliminate the influence of industry and year differences on the company’s dual innovation, we added industry and annual dummy variables into the model. All variables are showin in Table 3.

**Table 3:** Variable definitions.

Variable symbol	Measurement
Inc	The percentage of development capital expenditure to total assets at the beginning of the year
Rad	The percentage of research expenditures to total assets at the beginning of the year
Dom	Perform principal component regression on five indicators of successor’s work experience, age, education background, overseas study background, and gender to construct a comprehensive indicator
Imp	Proportion of control of listed companies owned by successors
Dis	Voting opinions other than "agree" are regarded as dissenting opinions
Age	Company age
Cas	Cash holdings
Gro	TobinQ
Ret	Annual return on assets
Pro	Institutional investor shareholding ratio
Rig	Year-end shareholders’ equity
Year	Take 2011 as the base year, and set dummy variables for the remaining years
Ind	Industry dummy variables defined based on the industry classification of the China Securities Regulatory Commission

## Empirical results

### Descriptive statistics

Table 4 is the descriptive statistics of the main variables in this paper. From the research samples, we can find that the average value of incremental innovation is 1.552, and the average value of radical innovation is 0.740, indicating that most of the investment in innovation activities of family businesses is used in the research phase, that is, it is mainly used for incremental innovation. The average implicit power of successors in the family business is 4.492, which indicates that successors nurtured by the previous generation of entrepreneurs is gradually taking the power; the average explicit power of the successors in the family business is 10.616, which indicates that the previous generation of entrepreneurs has already handed over some management power to their successors. The average board’s dissent is 0.029 indicating that in the 310 observation samples about 3% of the sample companies have board’s dissent.

**Table 4:** Descriptive statistics.

Variables	N	St.d	Mean	Min	Max
Inc	310	2.192	1.552	0.000	7.650
Rad	310	0.348	0.740	0.000	5.364
Dom	310	6.587	4.492	0.000	18.000
Imp	310	6.297	10.616	0.000	54.920
Dis	310	0.909	0.029	0.000	1.000
Age	310	8.557	6.033	0.000	26.000
Cas	310	4.59e+08	9.79e+08	-8.23e+08	7.09e+09
Gro	310	2.848	2.337	0.153	14.323
Ret	310	0.073	0.152	-1.951	0.296
Pro	310	5.77	6.451	.000	28.665
Rig	310	3.80+09	3.15+09	3.880e+08	2.82+10

### Regression results and analysis

Based on the hypothesis of this study, the following regression model is set to test hypotheses H1 and H2.


$${Inc}_{i,t}=a_{0}+a_{1}{Dom}_{i,t}+a_{2}\sum {Control}_{i,t}+\varepsilon_{i,t}$$ (1)

$${Inc}_{i,t}=a_{0}+a_{1}{Imp}_{i,t}+a_{2}\sum {Control}_{i,t}+\varepsilon_{i,t}$$ (2)

$${Rad}_{i,t}=a_{0}+a_{1}{Dom}_{i,t}+a_{2}\sum {Control}_{i,t}+\varepsilon_{i,t}$$ (3)

$${Rad}_{i,t}=a_{0}+a_{1}{Imp}_{i,t}+a_{2}\sum {Control}_{i,t}+\varepsilon_{i,t}$$ (4)

In order to test the hypothesis H3, this study introduces board’s dissent in the regression model to test the mediating effect. The measurement model is as follows:

$${Dis}_{i,t}=a_{0}+a_{1}{Dom}_{i,t}+a_{2}\sum {Control}_{i,t}+\varepsilon_{i,t}$$ (5)

$${Dis}_{i,t}=a_{0}+a_{1}{Imp}_{i,t}+a_{2}\sum {Control}_{i,t}+\varepsilon_{i,t}$$ (6)

$${Inc}_{i,t}=a_{0}+a_{1}{Dom}_{i,t}+{a_{2}{Dis}_{i,t}+a}_{3}\sum {Control}_{i,t}+\varepsilon_{i,t}$$ (7)

$${Inc}_{i,t}=a_{0}+a_{1}{Imp}_{i,t}+a_{2}{Dis}_{i,t}+a_{3}\sum {Control}_{i,t}+\varepsilon_{i,t}$$ (8)

$${Rad}_{i,t}=a_{0}+a_{1}{Dom}_{i,t}+{a_{2}{Dis}_{i,t}+a}_{3}\sum {Control}_{i,t}+\varepsilon_{i,t}$$ (9)

$${Rad}_{i,t}=a_{0}+a_{1}{Imp}_{i,t}+a_{2}{Dis}_{i,t}+a_{3}\sum {Control}_{i,t}+\varepsilon_{i,t}$$ (10)

Table 5 lists the partial regression results of research hypotheses H1 and H3. The results of Model 1 and Model 4 show that the explicit power of successors is positively correlated with incremental innovation (beta = 0.268, p<0.01), and the implicit power of successors is positively correlated with incremental innovation (beta = 0.457, p< 0.01). The regression standardization coefficient of explicit power and R^2^ are better than implicit power. This shows that explicit power has a stronger positive impact on incremental innovation than implicit power, which means hypothesis H1 is supported. At the same time, the results of Model 1—Model 6 show that board’s dissent plays a partial mediating role in the implicit power, explicit power and incremental innovation of successors.

**Table 5:** Incremental innovation model test results.

Variables	Inc	Dis	Inc	Inc	Dis	Inc
	Model 1	Model 2	Model 3	Model 4	Model 5	Model 6
Dom	0.268*** (2.76)	-2.266** (-2.47)	0.154* (1.68)			
Imp				0.457*** (5.02)	-3.05e+76** (-1.67)	1.838*** (5.94)
Dis			-1.970*** (-6.13)			-0.363*** (-4.22)
Age	-0.043** (-2.31)	-0.981 (-0.39)	-0.037** (-2.13)	-0.038** (-2.12)	-1.070 (-1.16)	-0.033* (-1.96)
Cas	0.000* (1.74)	-1.000 (-0.97)	0.000*** (2.69)	0.000** (2.01)	-1.000* (-1.90)	0.000*** (2.92)
Gro	0.136*** (2.94)	-0.997 (-0.02)	0.138*** (3.24)	0.115*** (2.62)	-1.057 (-0.35)	0.125*** (3.06)
Ret	0.4538 (0.73)	-2.948 (-0.99)	0.2185 (0.38)	0.669 (1.12)	-12.987 (-1.60)	0.375 (0.67)
Pro	0.037** (2.23)	-1.067 (-1.20)	0.0271* (1.76)	0.040** (2.50)	-1.037 (-0.72)	0.030** (2.00)
Rig	-0.000 (-1.41)	-1.000*** (-2.34)	-0.000 (-0.45)	-0.000 (-0.89)	-1.000 (-1.27)	-0.000 (-0.13)
_cons	0.085 (0.16)	-118.292*** (-3.16)	-2.063*** (-3.40)	0.089 (0.17)	-2.27e+47* (-1.75)	-1.875*** (-3.23)
Year	Control					
Ind	Control					
N	310	310	310	310	310	310
R^2^	0.200	0.217	0.317	0.259	0.397	0.361

***, **, and * are significant at the statistical level of 1%, 5%, and 10%, respectively. The data in brackets of Model 2 and Model 4 are Z value, and the data in brackets of other models are T value.The R^2^ in Model 2 and Model 4 is Pseudo R^2^, and the R^2^ in the other models is Adj R^2^.

Table 6 lists the partial regression results of research hypotheses H2 and H3. The results of Model 7 and Model 10 show that there is a positive correlation between the implicit power of successors and radical innovation (beta = 0.097, p<0.05), and the explicit power of successors and radical innovation are not correlated at the 10% level. This shows that compared with explicit power, implicit power has a stronger positive impact on incremental innovation, supporting H2. At the same time, the results of Model 7-Model 12 show that the directors’ dissent plays a part of the mediating effect in the implicit power of successors and radical innovation. However, it is worth noting that the explicit power of successors is positively correlated with board’s dissent and is significant at the 5% level. This shows that one of the reasons why there is no correlation between explicit power of successors and radical innovation is that there is a break between the board of directors dissent and radical innovation, that is, the relationship between the board of directors dissent and radical innovation is not significant. It is suggested that risky enterprise decisions such as radical innovation, the explicit power of successors does not promote executive directors to follow successors to engage in risky innovation activities, and such decisions are all made in informal meetings.

**Table 6:** Radical innovation model test results.

Variable	Rad	Dis	Rad	Rad	Dis	Rad
	Model 7	Model 8	Model 9	Model 10	Model 11	Model 12
Dom	0.097** (1.99)	-2.266** (-2.47)	0.070** (0.153)			
Imp				0.018 (0.38)	-3.05e+76** (-1.67)	0.045 (0.96)
Dis			-0.456*** (-2.66)			-.536*** (-3.13)
Age	-0.004 (-0.44)	-0.981 (-0.39)	-0.003 (-0.29)	-0.006 (-0.59)	-1.070 (-1.16)	-0.004 (-0.43)
Cas	-0.000** (-2.48)	-1.000 (-0.97)	-0.000** (-2.14)	-0.009** (-2.56)	-1.000* (-1.90)	-0.000** (-2.19)
Gro	0.033 (1.43)	-0.997 (-0.02)	0.034 (1.47)	0.000 (1.17)	-1.057 (-0.35)	0.030 (1.32)
Ret	0.213 (0.68)	-2.948 (-0.99)	0.158 (0.51)	0.027 (0.86)	-12.988 (-1.60)	0.182 (0.59)
Pro	-0.002 (-0.22)	-1.067 (-1.20)	-0.004 (-0.50)	-0.268 (-0.17)	-1.037 (-.72)	-0.004 (-0.53)
Rig	0.000*** (5.21)	-1.000** (-2.34)	0.000*** (5.66)	0.000*** (5.53)	-1.000** (-1.75)	0.000*** (6.02)
_cons	0.268 (1.26)	-118.292*** (-3.16)	-0.160 (-0.49)	0.249 (0.93)	-2.27e+47* (-1.75)	-0.324 (-1.01)
Year	Control					
Ind	Control					
N	310	310	310	310	310	310
R^2^	0.149	0.217	0.172	0.134	0.217	0.168

***, **, and * are significant at the statistical level of 1%, 5%, and 10%, respectively. The data in brackets of Model 2 and Model 4 are Z value, and the data in brackets of other models are T value.The R^2^ in Model 2 and Model 4 is Pseudo R^2^, and the R^2^ in the other models is Adj R^2^.

### Robustness test

When discussing the relationship between power and corporate innovation [10], some scholars did not consider the endogeneity problem, and therefore the endogeneity problem in this study is not serious. However, considering that there may be a lag in innovation input, this study takes a year lagging to deal with the dependent variable to solve the possible endogenous problem. Due to the one year lagging leading to some data missing, this study finally obtained complete data of 233 listed family companies and retested hypotheses. The results showed that the research conclusions of this study are still supported. The test results are shown in Table 7 below.

**Table 7:** Test results.

Variable	Inc Model						Rad Model					
	Inc	Dis	Inc	Inc	Dis	Inc	Rad	Dis	Rad	Rad	Dis	Rad
	Model 1	Model 2	Model 3	Model 4	Model 5	Model 6	Model 7	Model 8	Model 9	Model 10	Model 11	Model 12
Dom	0.368*** (3.19)	-2.9448 (-2.79)	0.212** (1.98)				0.147** (2.30)	-2.944*** (-2.79)	0.116* (1.84)			
Imp				0.444*** (4.31)	-6.722** (2.30)	0.308*** (3.20)				0.002 (0.04)	-6.722** (-2.30)	0.034 (0.57)
Dis			-2.071*** (-6.21)			-1.981*** (-6.05)			-0.343* (-1.74)			-.456** (-2.29)
Age	-0.016 (-0.73)	-1.099 (-1.43)	-0.024 (-1.23)	-0.024 (-1.17)	-1.061 (-0.99)	-0.028 (-1.49)	-0.002 (-0.16)	-1.099 (-1.43)	-0.003* (-0.28)	-0.008 (-0.70)	-1.061 (-0.99)	-0.009 (-0.78)
Cas	0.000* (1.86)	-1.000 (-0.62)	0.000*** (3.02)	0.000* (1.95)	-1.000 (-0.87)	0.000*** (3.08)	-0.000*** (-3.32)	-1.000 (-0.62)	0.000*** (-3.03)	-0.000*** (-3.39)	-1.000 (-0.87)	0.000*** (-3.03)
Gro	0.123** (2.40)	-1.055 (-0.34)	0.128*** (2.78)	0.103** (2.08)	-1.044 (-0.28)	0.116** (2.56)	0.024 (0.86)	-1.055 (-0.34)	-0.025 (0.90)	0.019 (0.67)	-1.044 (-0.28)	0.021 (0.78)
Ret	1.236 (0.81)	-3.23e+5** (-2.55)	-0.435 (-0.31)	2.90** (2.00)	2.91e+6*** (2.21)	0.685 (0.50)	0.839 (1.03)	-3.23e+5** (-2.55)	0.562 (0.68)	1.267 (1.56)	2.91e+6*** (2.21)	0.757 (0.91)
Pro	0.024 (1.26)	-1.087 (-1.18)	0.015 (0.87)	0.022 (1.22)	-1.085 (-1.22)	0.014 (0.86)	0.003 (0.27)	-1.087 (-1.18)	0.001 (0.12)	0.002 (0.23)	-1.085 (-1.22)	0.000 (0.05)
Rig	-0.000 (-1.52)	-1.000*** (-2.67)	-0.000 (-0.63)	-0.000 (-0.63)	-1.000 (-1.61)	-0.000 (-0.07)	-0.000*** (-4.93)	-1.000*** (-2.67)	1.000*** (5.18)	0.000*** (5.56)	-1.000 (-1.61)	0.000*** (5.83)
_cons	-0.395 (-0.66)	-41.507** (-2.37)	-2.069*** (-3.16)	-0.176 (-0.30)	-83.158*** (2.63)	-2.139*** (-3.46)	0.426 (1.30)	-41.507** (-2.37)	0.062 (0.16)	0.249 (0.76)	-83.158*** (-2.63)	-0.203 (-0.54)
Year	Control											
Ind	Control											
N	233	233	233	233	233	233	233	233	233	233	233	233
R^2^	0.210	0.298	0.358	0.246	0.330	0.382	0.196	0.298	0.206	0.170	0.330	0.191

***, **, and * are significant at the statistical level of 1%, 5%, and 10%, respectively. The data in brackets of Model 2 and Model 4 are Z value, and the data in brackets of other models are T value.The R^2^ in Model 2 and Model 4 is Pseudo R^2^, and the R^2^ in the other models is Adj R^2^.

To make the research more convincing, this research re-evaluates the implicit and explicit power of successors. The implicit power of the successor is measured by the political connection of the successor (1 for holding a position at a government authority and 0 for not), and the implicit power of the successor is measured by ownership. In this paper, the hypothesis is tested again according to this variable, and the regression results are consistent with the previous research which are shown in Table 8.

**Table 8:** Test results.

Variable	Inc Model						Rad Model					
	Inc	Dis	Inc	Inc	Dis	Inc	Rad	Dis	Rad	Rad	Dis	Rad
	Model 1	Model 2	Model 3	Model 4	Model 5	Model 6	Model 7	Model 8	Model 9	Model 10	Model 11	Model 12
Dom	0.302*** (2.98)	-3.20*** (-2.96)	0.174* (1.81)				0.277*** (5.81)	-3.201*** (-2.96)	0.256*** (5.28)			
Imp				0.459*** (5.09)	-2.22e+93* (-1.76)	0.308*** (3.20)				0.032 (0.69)	-2.22e+93* (-1.76)	0.059 (1.27)
Dis			-1.952*** (-6.06)			-1.838*** (-5.95)			-0.318* (-1.96)			-.545*** (-3.19)
Age	-0.054*** (-2.88)	-0.938 (-1.41)	-0.043** (-2.48)	-0.043** (-2.41)	-1.069 (-1.15)	-0.037** (-2.20)	-0.012 (-1.41)	-0.938 (-1.41)	-0.011 (-1.21)	-0.005 (-0.58)	-1.069 (-1.15)	-0.003 (-0.38)
Cas	0.000 (1.26)	-1.000* (-1.95)	0.000** (2.36)	0.000* (1.96)	-1.000* (-1.94)	0.000*** (2.88)	-0.000*** (-3.42)	-1.000* (-1.95)	-0.000*** (-3.06)	-0.000** (-2.58)	-1.000* (-1.94)	-0.000** (-2.19)
Gro	0.096** (2.07)	-0.917 (-0.66)	0.115*** (2.69)	0.122*** (2.78)	-1.054 (-0.33)	0.131*** (3.20)	0.006 (0.26)	-0.917 (-0.66)	0.009 (0.41)	0.027 (1.16)	-1.054 (-0.33)	0.029 (1.29)
Ret	0.646 (1.04)	-4.815 (-1.41)	0.332 (0.58)	0.701 (1.18)	-13.162 (-1.64)	0.401 (0.72)	0.300 (1.03)	-4.815 (-1.41)	0.249 (0.86)	0.264 (0.84)	-13.162 (-1.64)	0.175 (0.57)
Pro	0.029* (1.75)	-1.041 (-0.82)	0.023 (1.46)	0.041** (2.55)	-1.036 (-0.69)	0.031** (2.04)	-0.009 (-1.20)	-1.041 (-0.82)	-0.011 (-1.34)	-0.002 (-0.18)	-1.036 (-0.69)	-0.004 (-0.55)
Rig	-0.000 (-1.15)	-1.000** (-2.10)	-0.000 (-0.29)	-0.000 (-0.77)	-1.000 (-1.21)	-0.000 (-0.03)	0.000*** (5.63)	-1.000** (-2.10)	0.000*** (5.91)	0.000*** (5.50)	-1.000 (-1.21)	0.000*** (5.99)
_cons	0.193 (0.36)	-314.285*** (-3.53)	-1.981*** (-3.22)	0.138 (0.27)	-1.14e+57* (-1.82)	-1.833*** (-3.16)	0.559 (2.20)	-314.285*** (-3.53)	0.204 (0.66)	0.239 (0.89)	-1.14e+57* (-1.82)	-0.346 (-1.08)
Year	Control											
Ind	Control											
N	310	310	310	310	310	310	310	310	310	310	310	310
R^2^	0.205	0.250	0.319	0.261	0.402	0.364	0.251	0.250	0.261	0.135	0.261	0.171

***, **, and * are significant at the statistical level of 1%, 5%, and 10%, respectively. The data in brackets of Model 2 and Model 4 are Z value, and the data in brackets of other models are T value.The R^2^ in Model 2 and Model 4 is Pseudo R^2^, and the R^2^ in the other models is Adj R^2^.

This study uses dual innovation to measure the degree to which directors are willing to take risks with their successors. Some studies show that higher risk projects undertaken by enterprises will lead to greater fluctuations in future earnings and greater uncertainty in cash inflows. Therefore, the volatility of corporate earnings, namely the standard deviation of ROA, is adopted to measure the risk-taking situation [10]. At present, many literature take every three years as an observation period to examine the manager’s tenure condition and calculate the enterprise’s risk-taking level. Given the particularity of family business, this study takes the succession’s office period as an observation period, and finally obtains 773 effective samples. The regression results are more consistent with the previous research as shown in Table 9.


$$RiskT_{i}=\sqrt{\frac{1}{N-1}\sum_{1}^{N}{\left(Adjroa_{i,n}-\frac{1}{N}Adjroa_{i,n}\right)}^{2}}$$

**Table 9:** Test results.

Variable	Risk	Dis	Risk	Risk	Dis	Risk
	Model 1	Model 2	Model 3	Model 4	Model 5	Model 6
Dom	0.002*** (4.46)	-1.163** (-1.95)	0.002** (3.99)			
Imp				0.000 (0.69)	2.670 (2.28)	0.005 (2.51)
Dis			-0.013** (-2.30)			-0.019*** (-3.24)
Age	-0.000 (-0.86)	-0.976 (-0.52)	-0.000 (-0.66)	-0.000 (-1.11)	-0.971 (-0.67)	-0.000 (-1.00)
Cas	0.000 (1.64)	-1.000 (-0.85)	0.000* (1.86)	0.000 (1.36)	-1.000 (-1.01)	0.000 (1.62)
Gro	0.005*** (5.71)	-1.016 (-0.11)	0.005*** (5.74)	0.005*** (4.96)	-0.930 (-0.54)	0.005*** (5.25)
Ret	0.336*** (28.06)	-2.89e+12*** (-4.27)	0.327*** (26.11)	0.341*** (27.36)	-7.01e+13*** (-4.62)	0.327*** (25.42)
Pro	0.000** (2.42)	-1.061 (-1.16)	0.001** (2.22)	0.001** (2.45)	-1.086 (-1.54)	0.001** (2.12)
Rig	-0.000 (-0.97)	-1.000*** (-2.67)	-0.000 (-0.65)	-0.000 (-0.28)	-1.000 (-2.47)	0.000 (0.05)
_cons	0.016 (1.58)	-8.083 (-1.55)	0.004 (0.31)	0.021** (2.05)	-16.919** (-1.96)	0.001 (0.07)
Year	Control					
Ind	Control					
N	773	773	773	773	773	773
R^2^	0.278	0.370	0.322	0.234	0.398	0.297

***, **, and * are significant at the statistical level of 1%, 5%, and 10%, respectively. The data in brackets of other models are T value.

In this formula, $Adjroa_{i,n}=\frac{Ebitda_{i,n}}{Asset_{i,n}}-\frac{1}{X_{n}}\sum_{K=1}^{X}\frac{Ebitda_{k,n}}{Asset_{k,n}}$. EBITDA_i, n_ represents the Earnings Before Interest, Tax, Depreciation and Amortization of the current year, Asset_i,n_ represents the total assets at the end of the current year, and X represents the total number of enterprises in the industry in which Enterprise i is engaged.

## Conclusion

### Research conclusion

This study uses A-share listed Chinese family companies from 2011 to 2019 as a sample to conduct empirical tests on the proposed hypotheses, and draws the following main conclusions: (i) Compared with implicit power, explicit power of successors has a stronger positive effect on incremental innovation which is an innovation with less risk. Directors who are calculating their own benefits will cooperate with their successors to carry out incremental innovation activities. On one hand, since explicit power allows successors to control more enterprise resources, it can directly cooperate with successors to sign documents to promote incremental innovation. On the other hand, Implicit power requires a series of tedious evaluations to increase incremental innovation. Therefore, compared with implicit power, explicit power of successors has a stronger positive impact on incremental innovation. Therefore, compared with implicit power, explicit power of successors has a stronger positive impact on incremental innovation. (ii) Compared with explicit power, implicit power has a stronger positive impact on radical innovation. Radical innovation is a risky transformative activity, which means directors will reduce radical innovation activities in order to avoid risks and safeguard their own interests. The implicit power of successors can induce the voluntary risk-taking behavior of directors, while the explicit power weaken the following behavior of risk-taking. Therefore, compared with implicit power, explicit power of successors has a stronger positive impact on incremental innovation. (iii) Board dissent has a mediating effect on the relationship between explicit power and incremental innovation, explicit power and incremental innovation, and implicit power and radical innovation, but there is no mediating effect between explicit power and radical innovation. Since corporate decisions need to be voted on by the board of directors, the more powerful the successor is, the less opposition there will be from the board. Because incremental innovation is conventional innovation with less risk, such decisions are ultimately allowed to be made at a formal meeting (a board meeting). Thus, there is a significant correlation between board dissent and incremental innovation. Radical innovation, however, is an unconventional innovation with high risk, which means the final decision often needs to be made by successors and senior executives in informal meetings or in private. Therefore, radical innovation is not vetoed only when the successor has implicit power, that is, board dissent is significantly associated with incremental innovation. On the contrary, radical innovation is easy to be vetoed when the successor has explicit power, so that there is no significant relationship between board dissent and radical innovation.

**Fig 2:**
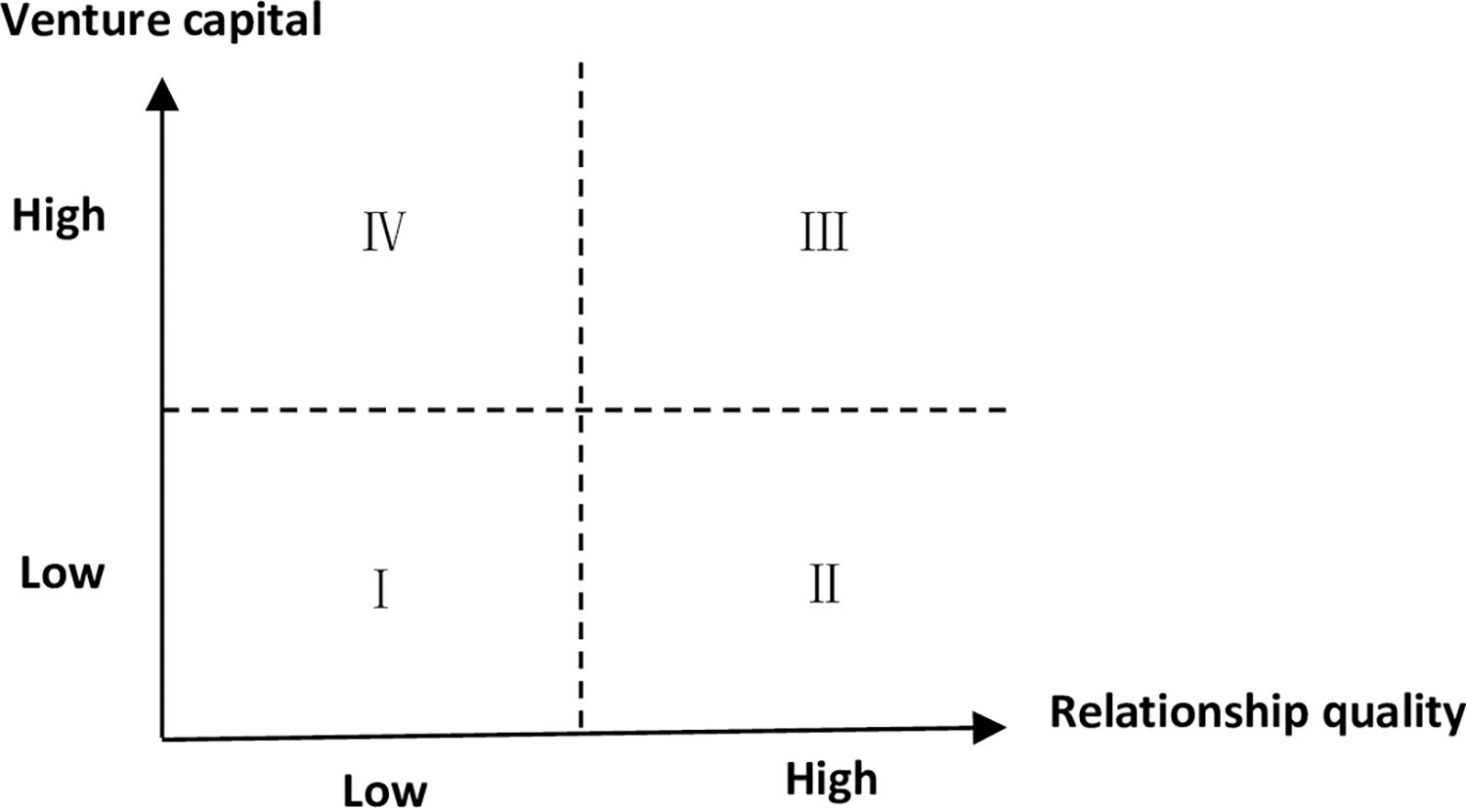
Relationship quality-venture capital situational matching.

Based on the research conclusions and existing literature research results, we construct a "relationship quality-venture capital situational matching" model in the context of social embeddedness between successors and executive members (as shown in Fig 2), and draws two general conclusions.

Firstly, explicit power provides a compulsory means to strongly influence others’ behavior, though it does not require psychological compliance. Implicit power acts on employees to produce voluntary compliance behavior, which is caused by spiritual belief, worship, identification and appreciation, rather than mandatory material means. It can be seen that explicit power mainly relies on compulsory means to maintain interpersonal relationship, which is of low quality in nature. The implicit power, however, mainly relies on psychological identity to maintain interpersonal relationship, which is essentially a high-quality relationship. As for the innovation, incremental innovation refers to the improvement, adjustment or extension of existing activities or technologies. It is often based on existing capabilities and technological development, and belongs to the continuous accumulation of innovation activities with less risk. On the contrary, radical innovation emphasizes the development of new products, new processes and new services. It is a subversion of the traditional technology and process, and is a replacement and a further development of the original market which is a transformative activity with high risks. Therefore, incremental innovation is of low investment risk in nature, while radical innovation is of high investment risk in nature.

Secondly, in the real situation, succession and directors in interpersonal interaction in the process of enterprise decision-making have four kinds of matching, namely Ⅰ low relationship quality—low investment risk, Ⅱ high relationship quality—low investment risk, Ⅲ high relationship quality—high investment risk, Ⅳ low relationship quality—high investment risk. Among them, the low Ⅰ relationship quality—low investment risk belongs to the golden mean situation in which members of the executive for successor investment decision-making will not interfere nor show positive support. Ⅱ relationship between high quality—low investment risk belongs to volunteer situation, where the directors for the successor investment decisions not only show the positive support, even help making investment decisions. Ⅲ high relationship quality—high investment risk belongs to the cooperation situation where the directors for the successor investment decisions showed positive support, but not for deeper direct investments. Ⅳ low relationship quality—high investment risk belongs to the opposite situation, in which the directors for the successor investment decisions will not interfere nor show positive support. Relevant studies also show that managers’ specific human capital and personal wealth are often highly dependent on the enterprises they work for, and they are unwilling to take risks for the sake of career concerns and personal interests. Therefore, in the low Ⅳ relationship quality—high risk investment situations, successor of the investment decision-making is the most difficult to realize.

### Research contribution

- The theoretical contributions of this research are as follows. Firstly, this study is a supplement to the literature on power and innovation, which reveals the differential effects of implicit power and explicit power on dual innovation. Although there have been studies on the influence of "single power on dual innovation" [12] or "different power on single innovation" [11], less literature has made an in-depth distinction between the two types of power and innovation. This study provides a good complement to the relationship between "different power" and "different innovation". Secondly, this study is based on the micro-perspective of interpersonal relationship between the successors of family business and directors. Previous scholars mainly studied the influence of power on innovation from the perspectives of "reputation" [38] and "social capital" [11], while this study shifted from the perspective of socially embedded power to the focus on the influence of interpersonal relationships on human behavior. This enriches the theoretical application of social embeddedness theory in the intergenerational succession of family business.
- This study has implications for other regions or countries: Firstly, business managers should be aware of the importance of corporate innovation. Family business is the oldest and most important organizational form in the history of human business, and it is also a modern organization with strong vitality and influence in contemporary economic society. However, the elimination rate of family businesses is quite high, which is closely related to the lack of effective sustainable development strategies for family businesses. As the source of economic growth, innovation is an important driving force for reshaping the competitive advantage of family enterprises and enhancing social value. Therefore, family business managers can increase the intensity of enterprise innovation from both capitalization expenditure development and expense expenditure. Secondly, managers should recognize the role of power in promoting innovation in enterprises, which is a very important element in the strategic decision-making process. When family businesses needs to continuously accumulate the ability to improve innovative activities, parent entrepreneurs should focus on the increase in the explicit power of successor managers to solve the problem of incremental innovation. On the other hand, when a family business is planning a radical innovation, the parent entrepreneur should focus on the cultivation of the implicit power of the successor manager.

### Limitations and prospects

The relationship between the succession power of family businesses, board dissent and corporate innovation is still in the exploratory stage of this study, and there are still many limitations. Firstly, the study sample does not cover family businesses in other regions or countries, and the reliability of the conclusions was still limited. In the future, the propositions of this study can be verified on the basis of the increase in the sample capacity of other regions or countries. Secondly, the research model in this paper can be further supplemented and improved. This study mainly explores the relationship between the implicit authority of successors and dual innovation from the perspective of the authority of interpersonal relations, and does not involve other aspects that affect interpersonal relationships (such as trust and norms). In future studies, the discussion of trust and norms can be added to obtain more applicable research conclusions.
