# Correction: The influence of successor’s implicit power and explicit power on dual innovation———The mediating effect of board dissent

**DOI:** 10.1371/journal.pone.0316655

**Published:** 2024-12-27

**Authors:** Wang Feifei, Wu Jiong, Marcello Russo, Luyang Gao

There is an error in affiliation 3 of author Marcello Russo. The correct affiliation 3 is: University of Bologna, Department of Management, and Bologna Business School, Bologna, Italy.
